# The mPEG-PCL Copolymer for Selective Fermentation of Staphylococcus lugdunensis Against Candida parapsilosis in the Human Microbiome

**DOI:** 10.4172/1948-5948.1000295

**Published:** 2016-06-19

**Authors:** Ming-Shan Kao, Yanhan Wang, Shinta Marito, Stephen Huang, Wan-Zhen Lin, Jon A Gangoiti, Bruce A Barshop, Choi Hyun, Woan-Ruah Lee, James A Sanford, Richard L Gallo, Yuping Ran, Wan-Tzu Chen, Chun-Jen Huang, Ming-Fa Hsieh, Chun-Ming Huang

**Affiliations:** 1 Department of Life Sciences, National Central University, Taoyuan, Taiwan; 2 Department of Dermatology, University of California, San Diego, CA, USA; 3 Surface Bioadvances Inc., San Diego, CA, USA; 4 Department of Pediatrics University of California, San Diego, CA, USA; 5 Department of Dermatology, Taipei Medical University, Taipei, Taiwan; 6 Department of Dermatology, West China Hospital, Sichuan University, Chengdu, China; 7 Department of Biomedical Engineering, Chung Yuan Christian University, Taoyuan, Taiwan; 8 Department of Biomedical Sciences and Engineering, National Central University, Taoyuan, Taiwan; 9 Moores Cancer Center; University of California, San Diego, CA, USA

**Keywords:** *C. parapsilosis*, Fermentation, Microbiome, Probiotic, *S. lugdunensis*

## Abstract

Many human skin diseases, such as seborrheic dermatitis, potentially occur due to the over-growth of fungi. It remains a challenge to develop fungicides with a lower risk of generating resistant fungi and non-specifically killing commensal microbes. Our probiotic approaches using a selective fermentation initiator of skin commensal bacteria, fermentation metabolites or their derivatives provide novel therapeutics to rein in the over-growth of fungi. *Staphylococcus lugdunensis* (*S. lugdunensis*) bacteria and *Candida parapsilosis* (*C. parapsilosis*) fungi coexist in the scalp microbiome. *S. lugdunensis* interfered with the growth of *C. parapsilosis* via fermentation. A methoxy poly(ethylene glycol)-*b*-poly*(ε*-caprolactone) (mPEG-PCL) copolymer functioned as a selective fermentation initiator of *S. lugdunensis*, selectively triggering the *S. lugdunensis* fermentation to produce acetic and isovaleric acids. The acetic acid and its pro-drug diethyleneglycol diacetate (Ac-DEG-Ac) effectively suppressed the growth of *C. parapsilosis in vitro* and impeded the fungal expansion in the human dandruff. We demonstrate for the first time that *S. lugdunensis* is a skin probiotic bacterium that can exploit mPEG-PCL to yield fungicidal short-chain fatty acids (SCFAs). The concept of bacterial fermentation as a part of skin immunity to re-balance the dysbiotic microbiome warrants a novel avenue for studying the probiotic function of the skin microbiome in promoting health.

## Introduction

The skin is colonized by a diverse array of microorganisms including bacteria and fungi. The skin microbiome is defined as the collection of all microbes that colonize the skin [1]. Environments at various topographical areas of skin can affect the microbial colonization. The major bacterial-fungal populations colonizing human scalps have been characterized [2,3]. Furthermore, the dysbiosis of bacterial-fungal populations has been implicated in scalp dandruff, which presents as significant problems to large numbers of people [4]. Dandruff scalps are associated with a higher abundance of *Malassezia restricta* and Staphylococcal species [5]. The severity of dandruff can range from mild scale formation similar to dry skin to seborrheic dermatitis [6]. The impairment of proper hydration in skin barrier can result in the typical epidermal proliferation, keratinocyte differentiation and stratum corneum maturation, which may cause dandruff [7]. Excessive secretion of the sebaceous gland also can underlie dandruff development. Besides dysfunction of the skin barrier and sebaceous gland, fungal/bacterial dysbiosis may be one of the factors that result in the progression of human dandruff.

Bacterial interference, or bacteriotherapy, in which commensal bacteria as probiotics are used to rein in the over-growth of opportunistic microbes, has been shown to be a promising modality for normalization of dysbiosis in the human microbiome [8–14]. Our pioneer studies demonstrated that the skin commensal bacteria can function as probiotic bacteria to undergo fermentation and produce short-chain fatty acids (SCFAs) [15]. *Staphylococcus epidermidis* (*S. epidermidis*), a skin commensal bacterium, can exploit glycerol fermentation to restrain the over-growth of opportunistic *Propionibacterium acnes* (*P. acnes*) [16]. Succinic acid, one of the SCFAs in fermentation products of *S. epidermidis*, inhibits the *P. acnes* growth *in vitro* and *in vivo*. Based on the data from our previous studies, we conjecture that production of SCFAs by probiotic bacteria in skin is a part of innate immunity to equilibrate the dysbiosis of the skin microbiome. In fact, it has been documented that SCFAs**,** although concentrations are relatively low in the skin, played a crucial role in altering the predominant residence of bacteria on normal human skin [17]. Many SCFAs with potent antimicrobial activities have been approved by the U.S. Environmental Protection Agency (EPA) as active ingredients for use as fungicides and bactericides on stored grains, poultry litter, and drinking water for poultry and livestock [18,19]. Furthermore, the Food and Drug Administration (FDA) has approved many SCFAs as flavor enhancers, miscellaneous and general purpose food chemicals, neutralizing agents, and pH control agents [20].

Currently, fungicides are the most effective drugs for treating dandruff. The zinc pyrithione (ZPT), selenium sulfide, coal tar and ketoconazole have been approved by the FDA to improve the dandruff by removal of fungi [7]. Keratolytic agents such as salicylic acid and sulfur were used to loosen the attachments between the corneocytes, allowing dandruff to get washed off [21]. However, the literature increasingly demonstrates that the prolong use of fungicides for treating a fungal infection of the scalp can be highly toxic [22]. The side-effects of keratolytic agents include dryness and irritation.

In the present study, we have identified and isolated *Staphylococcus lugdunensis* (*S. lugdunensis*) bacterium and *Candida parapsilosis* (*C. parapsilosis*) fungus from human dandruff flakes. We also demonstrated that *S. lugdunensis* can counteract the growth of *C. parapsilosis* via fermentation, validating the probiotic activity of *S. lugdunensis.* It has reported that bacteria can use a polyethylene glycol (PEG) polymer as a carbon source and fermentatively convert PEG to acetate and ethanol [23]. A diblock methoxy poly(ethylene glycol)-*b*-poly(*ε*-caprolactone) (mPEG-PCL) polymer was synthesized as a selective fermentation initiator which can exclusively trigger the fermentation of *S. lugdunensis*, but not *C. parapsilosis,* to yield acetate. We also revealed that acetate and its analog exert excellent activities against the growth of fungi in human dandruff flakes.

A prevalence of dandruff of up to 50% was found in the general population and approximately 50 million people suffer from dandruff in the United States (U.S.) with nearly $300 million spent on various dandruff treatment products annually [24]. Thus, the significance in this study includes providing a brand new approach to treat the fungal infection of the scalp skin, thereby benefiting the entire community of patients with dandruff or seborrheic dermatitis. Besides dandruff, hyperalimentation solutions, prosthetic devices, indwelling catheters and the nosocomial spread of disease through the hands of health care workers can be caused by *C. parapsilosis* infections [25]. Therefore, fermenting *S. lugdunensis* bacteria and their ferment metabolites may be novel therapeutics for the treatment of *C. parapsilosis*-associated infections.

## Materials and Methods

### Dandruff collection and microbial growth

Ethical approval for dandruff collection was obtained from Department of Dermatology, Taipei Medical University, Taiwan. The written consents from all participants were obtained before conducting dandruff collection. Those dandruff flakes with sizes greater than 1 mm^2^ were placed on malt extract agar (MEA) (Scharlab, S.L., Barcelona, Spain) plate’s right after dandruff collection. Agar plates with dandruff flakes were incubated at 30°C until the microbial colonies were formed.

### Microbial identification

Colonies on MEA plates were picked up by sterile toothpicks and DNA was extracted by an EasyPure Genomic DNA Spin kit (Bioman Scientific Co., Ltd, Taipei, Taiwan). For bacterial identification, polymerase chain reaction (PCR) with 16S rRNA 27F and 534R primers in addition to sequencing of PCR products was conducted as previously described [26]. For fungal identification, the D1/D2 5.8S rRNA gene was sequenced directly from the PCR products by using the primer pairs ITS1-Reverse (5’-TCCGTAGGTGAACCTGCGG-3’) and ITS4-Forward (5’-TCCTCCGCTTATTGATATGC-3’) [27]. PCR was performed under the following conditions: after an initial 3 min denaturation step at 95°C, 28 cycles of amplification were performed, each including 30 sec denaturation at 95°C, 30 sec annealing at 52°C and 1.5 min extension at 72°C, followed by a final 5 min extension at 72°C. The gene sequences of both 16S rRNA and D1/D2 5.8S rRNA were analyzed using the basic local alignment search tool (BLASTn).

### Culture of microbes

Identified bacteria and fungi were cultured in tryptic soy broth (TSB) and potato dextrose agar (PDA) (Sigma, St. Louis, MO, USA), respectively. Overnight cultures were diluted 1:100 and cultured to an absorbance at 600 nm [optical density (OD)_600_]=1.0. For some experiments, microbes were harvested by centrifugation at 5,000 g for 10 min, washed with phosphate buffered saline (PBS), and suspended in PBS.

### Co-culture of *S. lugdunensis* and *C. parapsilosis*

*S. lugdunensis* [10^5^ colony forming unit (CFU)] was co-cultured with *C. parapsilosis* (10^5^ CFU) in rich media (10 ml) [10 g/l yeast extract (Biokar Diagnostics, Beauvais, France), 3 g/l TSB, 2.5 g/l K_2_HPO_4_ and 1.5 g/l KH_2_PO_4_] in the presence or absence 20 g/l glycerol. After 3-day culture, media containing the microbes with a serial dilution (1–10^5^ CFU in 10 μl H_2_O) were spotted on furazolidone (10 μg/ml; Sigma)- containing PDA plates for 3 days.

### Synthesis and characterizations of mPEG-PCL

The mPEG-PCL diblock polymer was synthesized by ring-opening polymerization of e-caprolactone (Sigma). Monomer e-caprolactone (0.308 moles) was introduced in round-bottom flask along with macro initiator methoxy poly(ethylene glycol) [mPEG, molecular weight (Mw=550, 3.96 mmoles) under purging of nitrogen gas. The mixture was then heated up. When the temperature reached 130°C, the catalyst stannous 2-ethyl hexanoate (Sigma) (0.272 mmoles) was added into the flask for 5 h. The product was firstly dissolved in dichloromethane and then precipitated in ether/hexane of a volumetric ratio of 7:3 for three times. The precipitated samples were collected and vacuum dried. The Mw of the polymer was measured by gel permeation chromatography (GPCmax VE2001, Viscotek, Texas, USA) connected to a refractive index detector (VE3580, Viscotek, Texas, USA). Two columns (500 and 1000 angstroms, American Polymer Standards Corporation, USA) were maintained at 40°C. The polystyrene standards (Mw=972, 6,480, 9,000, and 18,200, Polymer Standards Service GmBH, German) were used to create the calibration line for the determination of Mw. The mPEG-PCL polymer was characterized by Fourier-transform Infrared spectroscopy (FT-IR) (FT-IR 410, JASCO, Tokyo, Japan) for the functional groups in the molecular structure of the polymer. The powdery mPEG-PCL polymer was compressed into a potassium bromide (KBr) plate for FT-IR measurements. The melting point which is related to Mw was measured by differential scanning calorimetry (DSC) (Jade DSC, Perkin-Elmer, Waltham, USA).

### Microbial fermentation

*S. lugdunensis* and *C. parapsilosis* (10^5^ CFU/ml) isolated from human dandruff flakes was incubated in rich media in the absence and presence of 20 g/l glycerol, 0.0005% mPEG diluted in water, or 0.0005% mPEG-PCL dissolved in 0.5% acetone at 37°C. Controls include rich media (with/without acetone) plus glycerol, mPEG, or mPEG-PCL without microbes. The 0.002% (w/v) phenol red (Sigma) in rich media with glycerol, mPEG or mPEG-PCL served as an indicator, changing the color from red-orange to yellow due to fermentation. The color change of phenol red from red-orange to yellow was monitored by the decrease in OD at 560 mM (OD_560_).

### Gas chromatography-mass spectrometry (GC/MS)

*S. lugdunensis* (10^5^ CFU/ml) was incubated in rich media in the presence of mPEG-PCL (0.0005%) for three days. After removing *S. lugdunensis* by centrifugation at 5,000 g for 10 min, SCFAs in the fermentation media (0.5 ml) were determined by ethyl acetate (Residue Analysis OmniSolv, EMD Millipore, Billerica, MA) liquid-liquid extraction after addition of the internal standard (0.1 mg/ml of ^2^H_7_- butyric acid, C/D/N Isotopes, Quebec, Canada), acidification with 0.5% ortho-phosphoric acid (Thermo Fisher Scientific, Fair Lawn, NJ) and saturation with sodium chloride (Thermo Fisher Scientific) followed by GC-MS analysis using an Agilent 5890 Series II GC coupled with 5971 MS detector (Agilent Technologies, Inc., Palo Alto, CA) [28]. A 70 eV electron was used for ionization. Acetic, propionic, isobutyric, butyric, isovaleric and valeric acids were quantified by a calibration curve made from six non-zero levels using the Free Fatty Acids Test Standard (Restek Corporation, Bellefonte, PA).

### Fungicidal effects of acetic acid and diethyleneglycol diacetate (Ac-DEG-Ac)

To determine the fungicidal activities of acetic acid and Ac-DEG-Ac, a pro-drug with two Ac esterified to a diethylene glycol (DEG) liner, *C. parapsilosis* (10^8^ CFU in 1 ml H_2_O) was incubated overnight with acetic acid in H_2_O (1 ml) or Ac-DEG-Ac in 4% dimethyl sulfoxide (DMSO) (1 ml) at various concentrations (0.01–500 mM) as indicated in each individual experiment in media in an eppendorf. The controls were kept in 1 ml H_2_O or 4% DMSO. After incubation, fungi were diluted 1:10–1:10^5^ with H_2_O. The percent growth inhibition of *C. parapsilosis* by acetic acid or Ac-DEG-Ac relative to fungi treated with control was determined.

### *Ex vivo* efficacy of acetic acid and Ac-DEG-Ac against fungi

The human dandruff flakes (>1 mm^2^) were cut in two halves, and the first half was incubated with acetic acid (10 mM in H_2_O) or Ac-DEG-Ac (10 mM in 4% DMSO) for 3 h at room temperature. The other half was incubated with H_2_O or 4% DMSO as a control. Dandruff flakes were placed on MEA plates and incubated for 4 days at 30°C. The change in the area (mm^2^) of fungal growth in dandruffs was measured and calculated with ImageJ software (NIH, Bethesda, MD, USA). More than three dandruff flakes per group experiment were used.

### Statistical analysis

To determine significances between groups, comparisons were made using the two-tailed *t*-test. For all statistical tests, the *P*-values of <0.05 (*), <0.01 (**), and <0.001 (***) were accepted for statistical significance.

## Results

### Inhibition of *C. parapsilosis* growth by glycerol fermentation of *S. lugdunensis*

**Figure 1:**
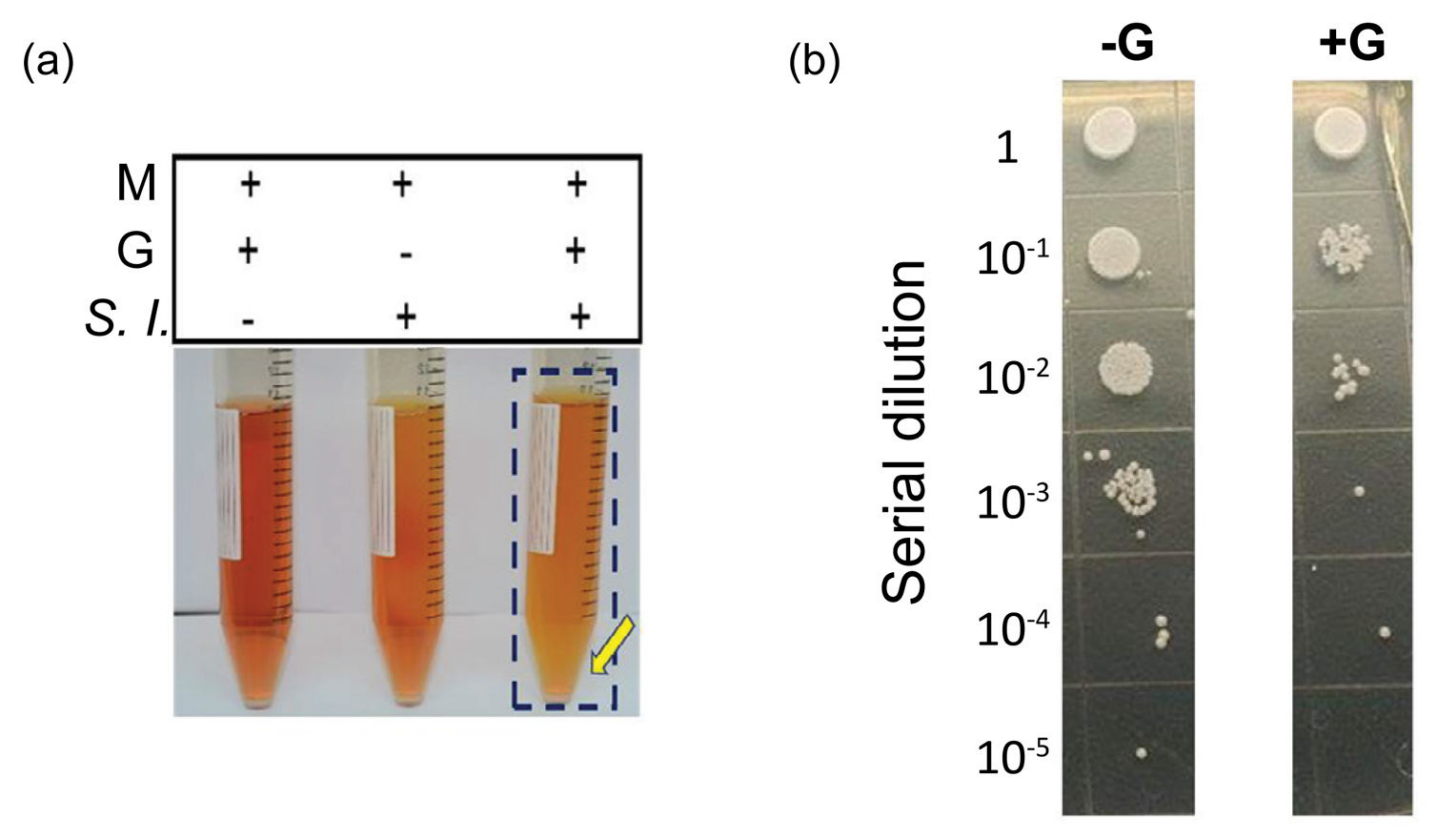
Probiotic activity of *S. lugdunensis* fermentation against *C. parapsilosis*. (a) *S. lugdunensis* (*S. l.*) (10^5^ CFU/ml), was incubated in rich media (M) with or without glycerol (G). Rich media plus glycerol without *S. lugdunensis* was included as a control. *S. lugdunensis* fermentation on 36 h was displayed. A color change to yellow in the media (marked in a blue frame and yellow arrow) indicates that bacterial fermentation has occurred. (b) *S. lugdunensis* (10^5^ CFU) was co-cultured with *C. parapsilosis* (10^5^ CFU) in rich media (10 ml) in the presence (+G) or absence (-G) of glycerol (20 g/l). After 3 day culture, media (10 μl) with a serial dilution (1–10^5^) were spotted on furazolidone (10 μg/ml) containing PDA plates for three days. Data representative of three separate experiments are shown.

Four bacteria (*S. lugdunensis*, *S. epidermidis*, *Staphylococcus warneri* and *Staphylococcus capitis*) and two fungi (*C. parapsilosis* and *Penicillium citrinum*) were identified and isolated from human dandruff flakes (Supplementary Table 1). To examine the fermentation activities of these bacteria and fungi, each individual microbe was incubated in rich media in the presence of 20 g/l glycerol, a naturally occurring metabolite found in human skin [29], as the carbon source. Rich media plus glycerol and rich media plus microbes were used as controls. To observe the fermentation process, rich media were added with phenol red, a fermentation indicator, to assess SCFA production as a result of glycerol fermentation. Consistent with our previous study [16], media in the culture of *S. epidermidis* with glycerol turned yellow which is the result of acid production three days following incubation (data not shown), demonstrating microbial fermentation. Besides *S. epidermidis*, the *S. lugdunensis* (Figure 1a) and *C. parapsilosis* (Supplementary Figure 1) were two microbes in human dandruff that can elicit fermentation of glycerol, making media turn yellow 36 and 96 h after incubation, respectively. *S. lugdunesis* is a normal inhabitant of the human skin. Previous studies demonstrated the isolation of *C. parapsilosis* from the human scalps and grew this fungus out along hair shafts planted in primary isolation media [30]. Our results here endorse that *S. lugdunesis* and *C. parapsilosis* co-exist in the human dandruff.

To investigate if *S. lugdunesis* fermentation influences the growth of *C. parapsilosis*, *S. lugdunesis* was co-cultured with *C. parapsilosis* in the presence or absence of glycerol for three days. To establish a *C. parapsilosis*-selective plate, media (10 μl) from the co-culture of *S. lugdunesis* and *C. parapsilosis* was spotted on a PDA plate supplemented with 10 μg/ml furazolidone. We found that 10 μg/ml furazolidone can completely kill *S. lugdunesis* without affecting the growth of *C. parapsilosis* (Supplementary Figure 2). Three days after microbial co-culture with/without glycerol, media with serial dilutions (1–10^5^) were spotted on *C. parapsilosis*-selective plates. The numbers of *C. parapsilosis* in the co-culture in the absence of glycerol found were at least one log order of magnitude greater than those in the co-culture in the presence of glycerol (Figure 1b). These data in Figure 1 suggest that *S. lugdunesis* mediates the glycerol fermentation to hamper the growth of *C. parapsilosis.*

### Synthesis of mPEG-PCL as a selective fermentation initiator of *S. lugdunensis*

**Figure 2:**
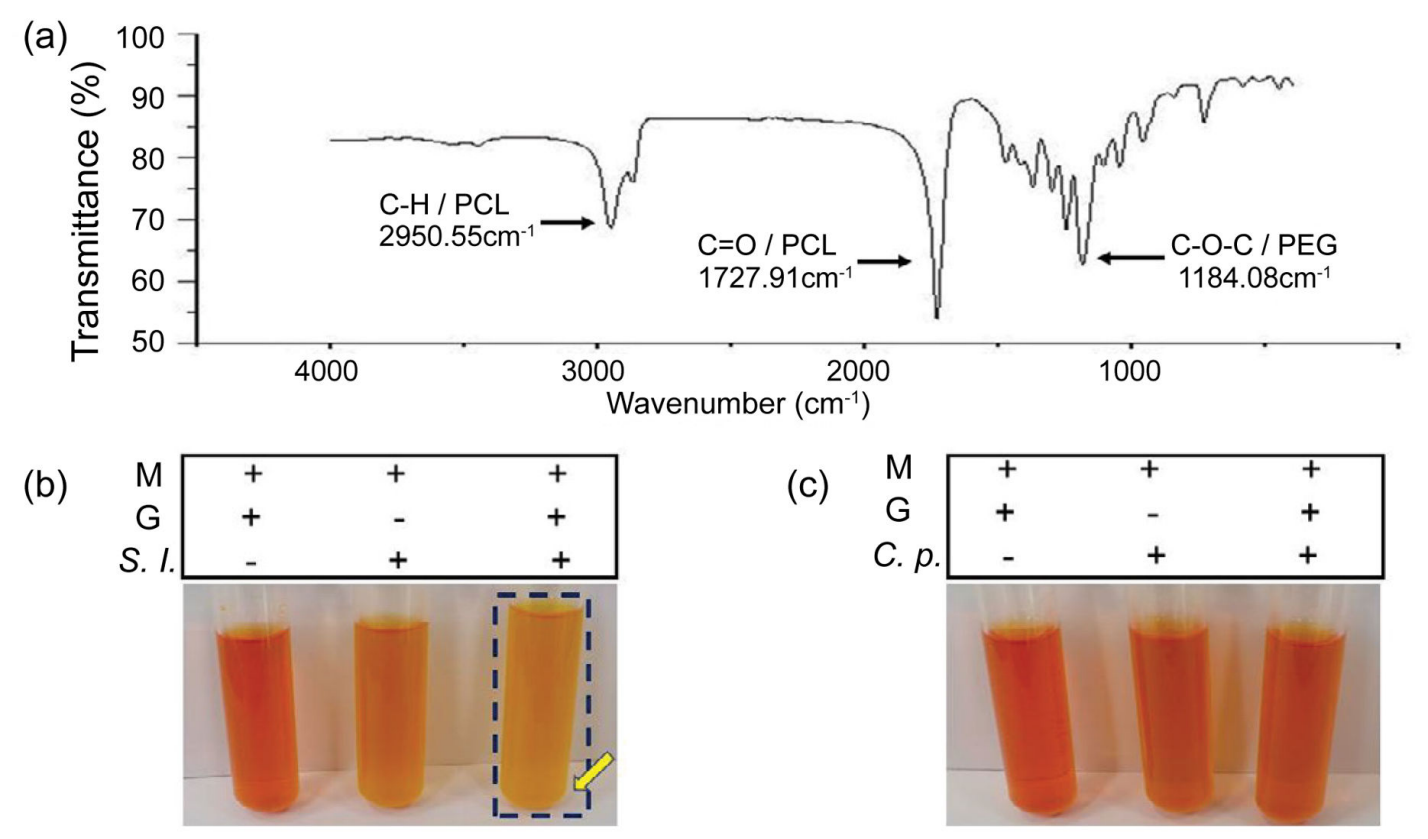
Characterizations of mPEG-PCL as a selective fermentation initiator of *S. lugdunensis* fermentation. (a) Three functional groups (C-O-C/ PEG/1184.1 cm^−1^; C=O/PCL/1727.9 cm^−1^; and C-H/PCL/2950.6 cm^−1^) of mPEGPCL were revealed in a FT-IR spectrum. (b) *S. lugdunensis*, but not (c) *C. parapsilosis*, fermented mPEG-PCL. 0.0005 % mPEG-PCL in 0.5 % acetone was used as a carbon source for fermentation. A blue frame and yellow arrow denote the mPEG-PCL fermentation of the *S. lugdunensis*. Representative data from three independent experiments are shown.

The polymerization was catalyzed by the addition of stannous 2-ethylhexanoate. Upon the complexation of monomer *ε*-caprolactone with the catalyst, the nucleophilic mPEG reacted with the monomer *ε*-caprolactone to give the product mPEG-PCL [31]. The purified polymer of mPEG-PCL was a white powder. The number-average Mw and weight-average Mw of PEG-PCL were 5,182 and 9,767 Da, respectively, leading to a polydispersity of 1.885. The functional groups revealed in a FT-IR spectrum were found at wave numbers of 1,727.9 cm^−1^ for C=O on PCL block and 1,184.1 cm^−1^ for C-O-C on mPEG block, respectively (Figure 2a). The melting point determined by DSC was at 53.6°C. Compared to our previous paper where the melting point of PEG-PCL having number-average Mw of 17,217 Da was 57.2°C, the mPEG-PCL used in the current study has lower Mw and yet lower melting point [32].

PEG-derived polymers have been employed as a carbon source for microbial fermentation [23]. Different microbial species make different enzymes that ferment specific substrates. To examine whether *S. lugdunesis* and *C. parapsilosis* differentially utilizes the mPEG or mPEG-PCL for fermentation, *S. lugdunesis* or *C. parapsilosis* was incubated in rich media in the absence or presence of 0.0005% mPEG-PCL. Controls include rich media with mPEG alone, mPEG-PCL alone or microbes alone. Incubation of *S. lugdunesis* with 0.0005% mPEG for 36 h did not induce fermentation (Supplementary Figure 3). As shown in Figure 2b, mPEG-PCL selectively triggered *S. lugdunesis*, but not *C. parapsilosis*, to undergo fermentation. In the culture of *S. lugdunesis* with mPEG-PCL, the phenol red-containing media started turning yellow 36 h after culture. The OD_560_/pH values of media with mPEG-PCL alone, *S. lugdunesis* alone or mPEG-PCL plus *S. lugdunesis* for 36 h are 0.53 ± 0.02/7.36 ± 0.01; 0.42 ± 0.01/7.12 ± 0.02; and 0.32 ± 0.01/6.88 ± 0.01. A significant decrease in OD_560_ and pH values in the media with mPEG-PCL plus *S. lugdunesis* indicated the mPEG-PCL fermentation of *S. lugdunesis*. No yellow media in the culture of *C. parapsilosis* with mPEG-PCL were detected. The mPEG-PCL was thus chosen as a selective fermentation initiator of *S. lugdunensis.* The exogenous addition of mPEG-PCL may be able to enhance the probiotic activities of *S. lugdunensis* for suppression of the growth of *C. parapsilosis* in human dandruff.

### Identification of SCFAs produced by mPEG-PCL fermentation by GC/MS

**Figure 3:**
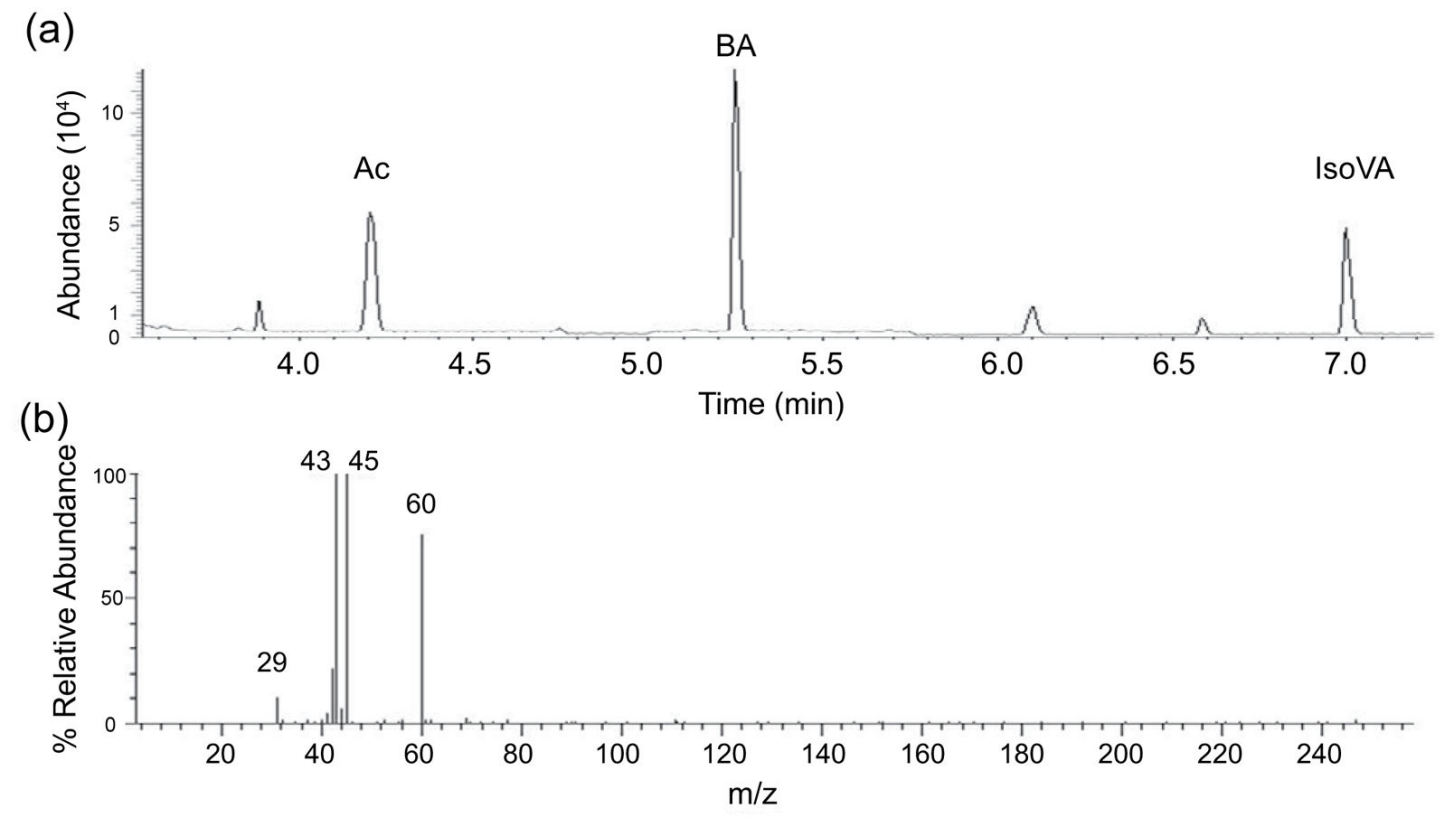
The ion chromatogram and mass spectrum from GC-MS for identification of SCFAs. (a) Total ion chromatogram for separation of the mixture of SCFAs containing acetic acid (Ac), ^2^H_7_-butyric acid (BA) (an internal standard) and isovaleric acid (IsoVa). (b) A mass spectrum for acetic acid. Molecular ions at 29, 43, 45 and 60 m/z for acetic acid were indicated.

To identify the SCFAs in the ferments, the *S. lugdunensis* were incubated in phenol red-free rich media in the presence of mPEG-PCL (0.0005%) for two days. Supernatants of bacterial culture *S. lugdunensis* mixed with ^2^H_7_-butyric acid (an internal standard) were subjected to GC-MS analysis. Two major SCFAs (acetic acid and isovaleric acid) in the fermented media of *S. lugdunensis* and ^2^H_7_-butyric acid were detected by GC separation (Figure 3a). Mass spectra of acetic acid (Figure 3b) and isovaleric acid (data not shown) were subsequently generated. Molecular ions at 29, 43, 45 and 60 m/z values corresponding to [HCO]^+^, [CH_3_CO]^+^, [CH_3_CO]^+^, CH_3_COOH for acetic acid are detected in a MS spectrum (Figure 3b). These results demonstrate that *S. lugdunensis* fermentatively metabolized mPEG-PCL into SCFAs.

### Suppression of the *C. parapsilosis* growth by acetic acid and Ac-DEG-Ac

**Figure 4:**
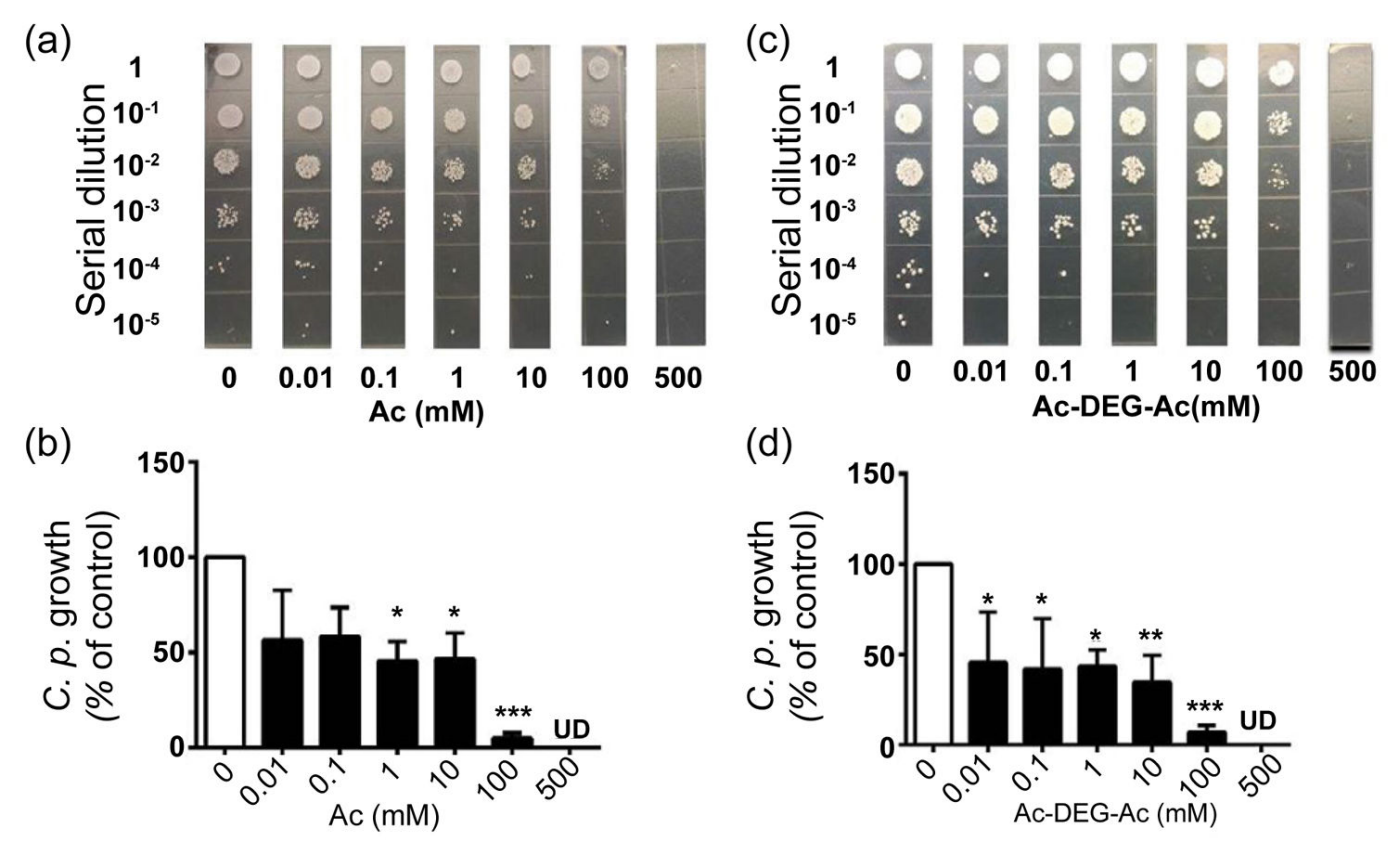
Suppression of *C. parapsilosis* growth by acetic acid and Ac-DEGAc. *C. parapsilosis* (10^8^ CFU) was incubated with 0.01–500 mM acetic acid in H_2_O (a, b) or Ac-DEG-Ac in 4% DMSO (c, d) overnight. Incubation of *C. parapsilosis* with H_2_O or 4% DMSO served as controls. After incubation, *C. parapsilosis* was diluted 1:10–1:10^5^ with H_2_O, and 10 μl of the dilutions were spotted on an agar plate. Percent growth inhibition of *C. parapsilosis* relative to the treatment with H_2_O control was presented. The CFU counts were illustrated as the mean ± standard derivation (SD) of six independent experiments (b, d). ***P<0.001; **P<0.01; *P<0.05, (two-tailed t-tests). UD, undetectable.

Two pharmacokinetic drawbacks of SCFAs as drugs are associated with their rapid metabolization and inability to accomplish effective concentrations *in vivo* [33,34]. Thus, Ac-DEG-Ac, a pro-drug of acetic acid (Ac) which contains two acetic acids esterified to a DEG linker, was included for evaluation of its fungicidal activity against *C. parapsilosis*. To compare Ac-DEG-Ac to acetic acid, *C. parapsilosis* was incubated with the concentrations (0.01–500 mM) of acetic acid (Figures 4a and 4b) in H_2_O or Ac-DEG-Ac (Figures 4c and 4d) in DMSO overnight. *C. parapsilosis* incubated with H_2_O or DMSO served as controls. Both acetic acid and Ac-DEG-Ac at concentrations in the range of 0.01– 10 mM inhibited approximately 50% growth of *C. parapsilosis.* Both agents at a concentration of 100 mM suppressed the growth of *C. parapsilosis* by greater than 90% and completely killed *C. parapsilosis* at a concentration of 500 mM, suggesting that both acetic acid and Ac- DEG-Ac exert anti-*C. parapsilosis* activities *in vitro*.

### Fungicidal activities of acetic acid and Ac-DEG-Ac against fungi in human dandruff

Filamentous microbes containing different fungi are typically found in human dandruffs. Besides anti-*C. parapsilosis* properties, we next examined if acetic acid and Ac-DEG-Ac can inhibit the growth of various fungi in human dandruffs. A human dandruff flake was cut in two halves, and the first half was incubated with 10 mM acetic acid or 10 mM Ac-DEG-Ac. The other half was incubated with H_2_O or DMSO as controls. Four days after incubation, we found that both acetic acid and Ac-DEG-Ac can efficiently block the extension of fungal growth in human dandruffs. The result suggests that acetic acid and Ac-DEG-Ac may have a broad spectrum of anti-fungal activity for treatment of human dandruff.

## Discussion

At least three new findings are presented in the current study. First, *S. lugdunensis* was found to be a probiotic bacterium which can ferment glycerol and mPEG-PCL. Second, the mPEG-PCL was used for the first time as a selective fermentation initiator to exclusively trigger the fermentation of *S. lugdunensis* against *C. parapsilosis*. The mPEG-PCL, PEG- or PCL-derived polymers can be developed as drug adjuvants in the future to reduce the amount of active drugs while maintaining the antimicrobial activity, decreasing the risk of generating resistant microbes. Third, the fungicidal activity of Ac- DEG-Ac demonstrates a successful approach for development of novel drugs from the resources of fermentation metabolites of skin probiotic bacteria. Yogurts containing live probiotic strains, the best examples of bacterial interference, have been used for centuries to maintain the digestive microbial ecosystem. Skin probiotic bacteria characterized in our previous papers can operate fermentation to suppress the pathogen colonization [15]. *S. lugdunensis*, a coagulase-negative staphylococcus, is a common colonizer of the human skin, and it is the only pathogen in about 10% of skin and soft tissue infections [35,36]. *C. parapsilosis*, a human opportunistic fungus, is frequently isolated from human skin [37]. It has been shown that *C. parapsilosis* can be isolated from human scalp [30]. The fungus has been recognized as commensal yeasts on the dog skin but also a causative microbe of seborrheic dermatitis, particularly in atopic dogs [38]. Although the clinical evidence about the oppositional relationship between *S. lugdunensis* and *C. parapsilosis* in the human skin is lacking, our data revealed that *S. lugdunensis* can exploit glycerol fermentation to hinder the growth of *C. parapsilosis in vitro* (Figure 1). During the development of dandruff, *S. lugdunensis* and *C. parapsilosis* may use the glycerol as a shared carbon source and produce different SCFAs as (antimicrobial) weapons to repel each other on the human scalp. *C. parapsilosis* may “win the battle” of microbial interference when the dandruff is persistent. Our strategy to cure dandruff is to deter the growth of *C. parapsilosis* by amplification of the probiotic activities of *S. lugdunensis* using a selective fermentation initiator.

PEG is a synthetic water-soluble polymer of the common structural formula H(OCH_2_CH_2_)_n_OH. An extracellular enzyme which can depolymerize long chain PEGs was identified [39]. The diol dehydratase and PEG acetaldehyde lyase in fermenting bacteria can degrade PEG [40]. However, results from other laboratories demonstrate that PEG degradation by fermenting bacteria was inhibited by ethylene glycol, probably owing to a blocking of the cellular uptake system. The results suggest that PEG was taken up into the bacteria and subsequently degraded inside [23]. The depolymerization of PEG can be catalyzed by bacteria via hydrolysis after a modification of the terminal ethylene glycol (EG) unit [23]. PEG fermentations yield acetaldehyde as an intermediate, which is further disproportionated to acetate and ethanol. A propionate-forming bacterium can ferment PEG to acetate and propionate [41]. PCL was found to be degraded by lipase from various sources and cutinase from a fungus [42,43]. PCL-degrading bacteria are widely distributed in nature. PCL depolymerase detected in both extracellular and intracellular fractions of bacterial cultures can efficiently degrade PCL [44]. No one reported that bacteria can use mPEG-PCL as a carbon source for fermentation. Our results demonstrate for the first time that *S. lugdunensis*, but not *C. parapsilosis,* can fermentatively metabolize mPEG-PCL to acetate and isovaleric acid (Figure 3). Since both *S. lugdunensis* and *C. parapsilosis* may use the same carbon source [e.g. glycerol; (Figure 1 and Supplementary Figure 1)] outcompete each other to survive. Although it is unclear why *S. lugdunensis* can fermeatatively metabolize mPEG-PCL, but not mPEG (Supplementary Figure 3), it is possible that mPEG-PCL containing more carbon atoms than mPEG provides a relatively abundant carbon source for *S. lugdunensis* fermentation. In addition, although incubation of *C. parapsilosis* with mPEG-PCL for 96 h did not induce fermentation (Figure 2c), it is worth investigate whether *C. parapsilosis* can non-fermentatively degrade mPEG-PCL to metabolites that affect the growth of *S. lugdunensis.* Our results support that the mPEG-PCL can function as a selective fermentation initiator to specially amplify the fermentation activity of *S. lugdunensis* against *C. parapsilosis.* Therefore, mPEG-PCL holds promise as a manipulation technique to reduce the fungal over-growth in the dysbiotic dandruff.

Phage therapy is yet another manipulation technique, by which selective overgrown microbes can be targeted with specific bacteriophages, thereby normalizing the dysbiotic skin microbiome. Although specific bacteriophages for *C. parapsilosis* have been not yet identified, the disadvantage of phage therapy includes the potential ability of bacteriophages to transfer the DNA from a microbe to another. This DNA transfer could be in charge of the transfer of pathogenicity determinants and virulence factors, resulting in the generation of even more resistant microbial strains [45–47]. Although the toxicity of mPEG-PCL to the skin has not been evaluated, the mPEG-PCL, a highly biocompatible and biodegradable copolymer, has been widely used as a component of nanoparticles for transdermal delivery [48]. Most importantly, both PEG and PCL have been approved by the FDA in specific applications used in humans as a drug delivery device. Thus, the development of mPEG-PLC as a targeted intervention may be relatively safe when it is employed to re-establish healthy patterns of bacterial-fungal interactions in the dysbiotic skin microbiome. Potentially, the mPEG-PCL can function as a drug adjuvant to augment the fermentation activity of skin probiotic bacteria and reduce the effective doses of fungicides in the future. The use of mPEG-PCL analog with lower dose of fungicides may minimize the risk of development of resistant fungi and the non-specific killing effect of fungicides on skin commensals.

**Figure 5:**
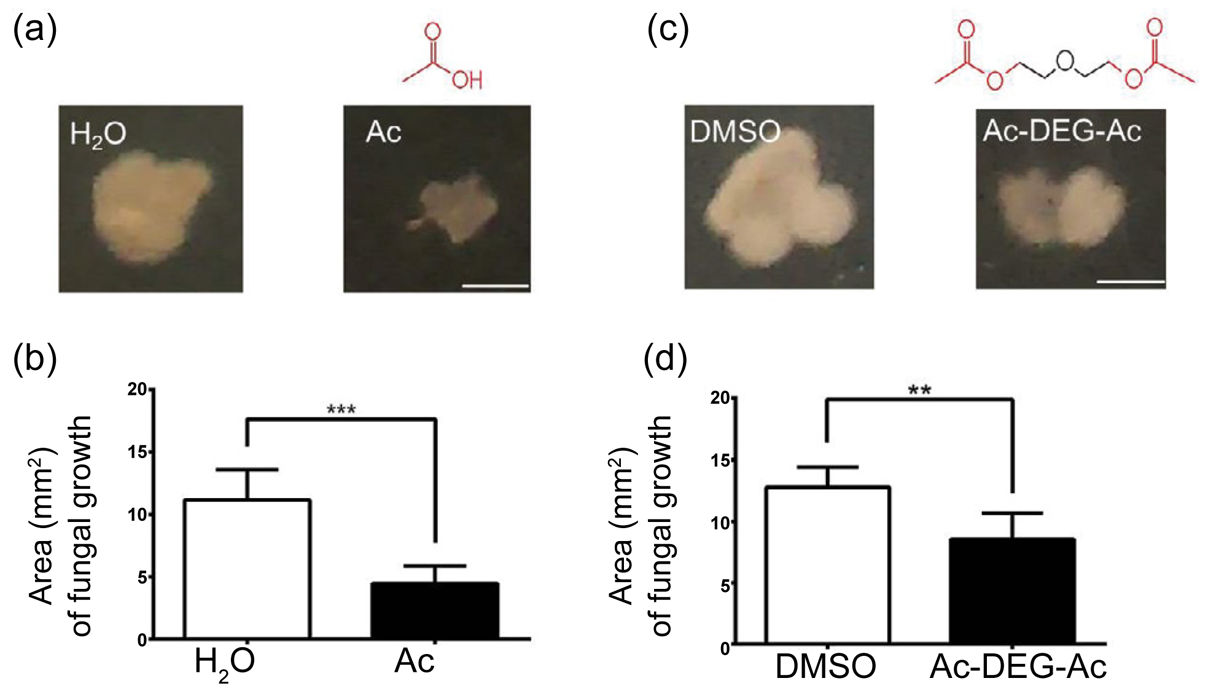
Inhibition of fungal growth in human dandruffs by **acetic acid and Ac-DEG-Ac.** A human dandruff flake was cut in half, and half was incubated with acetic acid in H_2_O (Ac) (a, b) or Ac-DEG-Ac (c, d) in DMSO for 3 h. The other half was incubated with H_2_O or DMSO as a control. Both chemical structures of acetic acid and Ac-DEG-Ac were illustrated. (b) The sizes (mm^2^) of fungal growth in dandruffs treated with acetic acid, Ac-DEG-Ac or their controls were quantified 4 days after placing dandruffs on MEA plates. At least three dandruff flakes per group experiment were used. ***P<0.001; **P<0.01, (two-tailed t-tests). Bars=2.0 mm.

The SCFAs produced by fermentation of intestinal microbes in the human colon can reach a high level in the 20–140 mM range that can effectively ward off local pathogens [49]. However, the amounts of SCFAs in peripheral circulation are relatively low, ranging from 3 to 7 μM [49]. Numerous SCFAs are frequently detected in the skin and in the secretions of skin glands, such as the sweat, but their concentrations are generally low. For example, sweat only contains 0.0096% acetic acid [50]. Although the levels of SCFAs in human scalp skin have yet to be quantified, it has been documented that SCFAs have short half-lives and the apparent difficulty of achieving pharmacologic concentrations when they are administered *in vivo*. Furthermore, although SCFA is a normal human metabolite and theoretically less toxic, SCFA at high doses may create an extremely acid solution, which may be toxic to skin cells. A butyric acid pro-drug named pivaloylomethyl butyrate (AN-9) has been developed to achieve an effective concentration of butyric acid [51]. To increase the half-life of propionic acid, we have synthesized a pro-drug of propionic acid by esterifying two active propionic acids to a DEG linker. The pro-drug of propionic acid exerts excellent antimicrobial activity against *S. aureus* [49]. In this study, we demonstrate that a pro-drug of acetic acid (Ac-DEG-Ac) that is relatively less acidic and contains two acetic acid moieties esterified to a DEG linker efficiently suppresses the growth of fungi in the human dandruff (Figure 5).

Esterases such as carboxylic esterase are capable of hydrolyzing the ester group of esterified pro-drug [52]. Although esterases are expressed by host cells, esterases in extracellular fluids or released from microbes have been identified [53–56]. Dead cells such as dandruff may also release esterases [57,58]. Future studies will determine if the Ac-DEG-Ac is cleaved by esterases released from microbes and/or dead cells to increase the local concentrations of acetic acids, thereby becoming pharmacologically effective to decolonize the fungi in dandruff flakes. We also cannot rule out the possibility of anti-fungal activity of uncleaved Ac-DEG-Ac. Although it remains unclear how SCFAs impede the fungal growth, prior findings suggested that a lowered intracellular pH value of microbes is a lethal mechanism of SCFA [59]. Mounting evidence has demonstrated the anti-inflammatory effect of SCFAs [60]. The anti-inflammatory effect of SCFAs may serve to promote immunological tolerance to commensal bacteria via stimulation of immunosuppressive interleukin (IL)10 production of regulatory T (T_reg_) cells, maximizing the probiotic activities of skin commensal bacteria to outcompete the over-growth of pathogens. Thus, it is worth exploring the impacts of acetic acid and Ac-DEG-Ac on the host inflammatory responses and long-term homeostasis of the human skin microbiome.

Lack of selectivity and induction of resistance are central problems for many fungicides. Vaccines, although the actions are fairly specific, may become less potent for treatments of patients with significant underlying health matters such as diabetes, surgical intervention, and immune suppression. In contrast, probiotic treatments, which are relatively independent on patients’ health matters, have little or no interference with commensal microbes may complement fungicides or vaccines for treatments of fungal infections in the skin.

## Conclusion

Here, we envision that the interference of bacteria with the growth of fungi via fermentation naturally occurs in the human scalp skin. The use of endogenous molecules, such as SCFAs in fermentation products, as natural fungicides is concordant with evolutionary medicine and provides a new set of tools for fighting the fungal resistance.

## Supplementary Material

Supple fig and table
