# Supplementary material for: The mPEG-PCL Copolymer for Selective Fermentation of Staphylococcus lugdunensis Against Candida parapsilosis in the Human Microbiome
Source: J Microb Biochem Technol. Author manuscript; Available in PMC 2017 Jan 18. (PMC5243119; doi:10.4172/1948-5948.1000295)
Supplement: Supple fig and table [file NIHMS818987-supplement-Supple_fig_and_table.pdf]

SUPPLEMENTARY MATERIALS

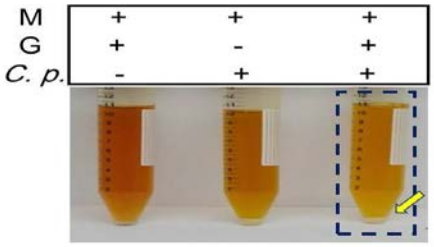

Supplementary Figure 1: The glycerol fermentation of *C. parapsilosis*. (a) *C. parapsilosis* (*C. p.*) (10^5 CFU/ml) was incubated in rich media (M) with or without glycerol (G). Rich media with glycerol alone serve as a control. The yellow media (a blue frame and yellow arrow) were observed four days after glycerol fermentation. Results are representative of three independent experiments.

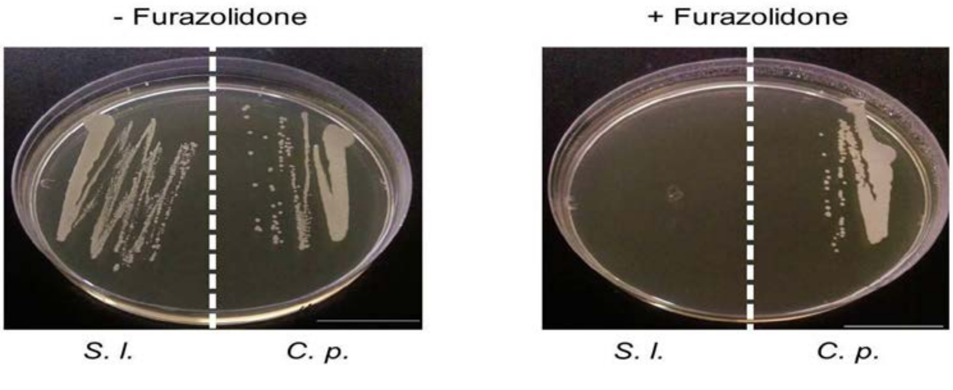

Supplementary Figure 2: Furazolidone inhibits the growth of *S. lugdunensis*, but not *C. parapsilosis*. *S. lugdunensis* (*S. l.*) or *C. parapsilosis* (*C. p.*) (10^7 CFU in 100 µl) was spread on MEA plates supplemented with (+) or without (-) 10 µg/ml furazolidone for three days. Bars=2.5 cm.

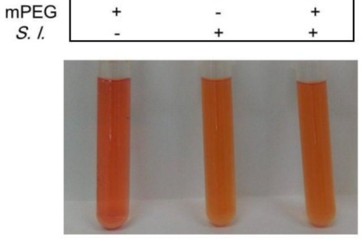

Supplementary Figure 3: No mPEG fermentation in *S. lugdunensis*. The *S. lugdunensis* (*S. l.*) bacteria (10^5 CFU/ml) were incubated in rich media (M) with or without 0.0005% mPEG (mPEG). Rich media with mPEG alone serve as a control. The OD560/pH values of media with mPEG alone, *S. lugdunesis* alone or mPEG plus *S. lugdunesis* for 36 h are 0.51 ± 0.01/7.32 ± 0.02; 0.41 ± 0.02/7.03 ± 0.01; and 0.43 ± 0.02/7.06 ± 0.02. No significant decrease in OD560 and pH values in the media with mPEG plus *S. lugdunesis* indicated that no mPEG fermentation occurred in the culture of *S. lugdunesis*. Results are representative of three separate experiments.

Supplementary Table 1: The sequences and percent identity of both 16S rRNA and D1/D2 5.8S rRNA genes of bacteria and fungi isolated from human dandruff.

Bacteria:

100% Identity with ***Staphylococcus lugdunensis*** HKU09-01, complete genome; NC_013893.1

CACGTAGTTAGCCGTGGCTTTCTGATTAGGTACCGTCAAGACGTGCACAG
TTACTTACACGTTTGTTCTTCCCTAATAACAGAGTTTTACGATCCTAAGAC
C TTCATCACTCACGCGGCGTTGCTCCGTCAGGCTTTCGCCCATTGCGGAA
GATTCCCTACTGCTGCCTCCCGTAGGAGTCTGGACCGTGTCTCAGTTCC
AGTGTGGCCGATCACCCTCTCAGGTCGGCTACGTATCGTTGCCTTGGTG
AGCCGTTACCTCACCAACTAGCTAATACGGCGCGGGTCCATCTATAAGTG
ATAGCAAAACCATCTTTCACTATTGAACCATGCGGTTCAACATATTATCCG
G
TATTAGCTCCGGTTTCCCGAAGTTGTCCCAGTCTTATAGGTAGGTTACCCA
CGTGTTACTCACCCGTCCGCCGCTAACGTCAAAGGAGCAAGCTCCTTATC
TGTTCGCTCGACTTGCATGTATTAGGCACGCCGCCAGCGTTCAT

100% Identity with ***Staphylococcus epidermidis*** ATCC 12228 chromosome, complete genome; NC_004461.1

CTGGCACGTAGTTAGCCGTGGCTTTCTGATTAGGTACCGTCAAGACGTGC
ATAGTTACTTACACATTTGTTCTTCCCTAATAACAGAGTTTTACGATCCGA
A GACCTTCATCACTCACGCGGCGTTGCTCCGTCAGGCTTTCGCCCATTGC
GGAAGATTCCCTACTGCTGCCTCCCGTAGGAGTCTGGACCGTGTCTCAG
TTCCAGTGTGGCCGATCACCCTCTCAGGTCGGCTACGCATCGTTGCCTT
GGTAAGCCGTTACCTTACCAACTAGCTAATGCGGCGCGGATCCATCTATA
A
GTGACAGCAAAACCGTCTTTCACTATTGAACCATGCGGTTCAATATATTAT

C
CGGTATTAGCTCCGGTTTCCCGAAGTTATCCCAGTCTTATAGGTAGGTTAT
CCACGTGTTACTCACCCGTCCGCCGCTAACGTCAGAGGAGCAAGCTCCT
CGTCTGTTCGCTCGACTTGCATGTATTAGGCACGCCGCCAGCGTTCATCC
TGAG

100% Identity with ***Staphylococcus warneri*** SG1, complete genome; NC_020164.1

CTGCTGGCACGTAGTTAGCCGTGGCTTTCTGATCAGGTACCGTCAAGATG
TGCACAGTTACTTACACATTTGTTCTTCCCTGATAACAGAGTTTTACGATC
C GAAGACCTTCATCACTCACGCGGCGTTGCTCCGTCAGGCTTTCGCCCAT
TGCGGAAGATTCCCTACTGCTGCCTCCCGTAGGAGTCTGGACCGTGTCT
CAGTTCCAGTGTGGCCGATCACCCTCTCAGGTCGGCTACGTATCGTTGC
CTTGGTAAGCCGTTACCTTACCAACTAGCTAATACGGCGCGGATCCATCT
A
TAAGTGACAGCAAAGCCGCCTTTCACTATTGAACCATGCGGTTCAATATG
T
TATCCGGTATTAGCTCCGGTTTCCCGAAGTTATCCCAGTCTTATAGGTAGG
TTATCCACGTGTTACTCACCCGTCCGCCGCTAACGTCAAAGGAGCAAGCT
CCTTATCTGTTCGCTCGACTTGCATGTATTAGGCACGCCGCCAGCGTTCA
TCCTGA

100% Identity with ***Staphylococcus capitis*** CR01 genomic scaffold, ScapHOJ_3778_scaffold7, whole genome shotgun sequence;
NZ_HG737339.1

TCAGGATGAACGCTGGCGGCGTGCCTAATACATGCAAGTCGAGCGAACA
GACGAGGAGCTTGCTCCTCTGACGTTAGCGGCGGACGGGTGAGTAACA
CGTGGATAACCTACCTATAAGACTGGGATAACTTCGGGAAACCGGAGCT
A
ATACCGGATAACATGTTGAACCGCATGGTTCAACAGTGAAAGACGGTCT
T
GCTGTCACTTATAGATGGATCCGCGCCGCATTAGCTAGTTGGTAAGGTAA
CGGCTTACCAAGGCAACGATGCGTAGCCGACCTGAGAGGGTGATCGGC
CACACTGGAACTGAGACACGGTCCAGACTCCTACGGGAGGCAGCAGTA
GGGAATCTTCCGCAATGGGCGAAAGCCTGACGGAGCAACGCCGCGTGA
GTGAAGAAGGTCTTCGGATCGTAAAACTCTGTTATTAGGGAAGAACAAA
T
GTGTAAGTAACTATGCACGTCTTGACGGTACCTAATCAGAAAGCCACGG
C TAACTACGTG

Fungi:

100% Identity with ***Candida parapsilosis*** strain YF005 18S ribosomal RNA gene, partial sequence; internal transcribed spacer 1, 5.8S ribosomal RNA gene, and internal; KR089865.1

ATGCTTAAGTTCAGCGGGTAGTCCTACCTGATTTGAGGTCGAATTTGGAA
GAAGTTTTGGAGTTTGTACCAATGAGTGGAAAAAACCTATCCATTAGTTT
A
TACTCCGCCTTTCTTTCAAGCAAACCCAGCGTATCGCTCAACACCAAACC
CGAGGGTTTGAGGGAGAAATGACGCTCAAACAGGCATGCCCTTTGGAAT
ACCAAAGGGCGCAATGTGCGTTCAAAGATTCGATGATTCACGAATATCTG
CAATTCATATTACTTATCGCATTTCGCTGCGTTCTTCATCGATGCGAGAAC
C
AAGAGATCCGTTGTTGAAAGTTTTGACTATTAAATAATCGGTTGACATTAA
A
TAAAATTTGGTTGAGTTTAATCTCTGGCAGGCCCCATATAGAAGGCCTACC
AAAGCAAAGTTTTCAAAAAAAGAAAAACACATGTGTAAGAAAAAATGCA
GT TAAGCACTTTTCATTCTGTAATGATCCTTCC

100% Identity with ***Penicillium citrinum*** isolate Bankole Paul 14-PC 28S ribosomal RNA gene, partial sequence; KJ880094.1

AGACGGGTCGCTTACGACCATTATGCCAGCGTCCGAGCCGAAGCGCGTT
CCTCGGTCCGGGCAGGCCGCATGGCACCCCTGGCTATAAGACGCCCCG
AGAGGCGTTACCTTCCAGAGGCCTTTGACCGGCCGCCCAAACCGACGCT
GGCCCGCCCGCGGGGAAGTACACCGGCACGAGTGCCGGCTGAACCCC
GCGGGCGAGTCTGGTCGCAAGCGCTTCCCTTTCAACAATTTCACGTGCT
GTTTAACTCTCTTTTCAAAGTGCTTTTCATCTTTCGATCACTCTACTTGTGC
GCTATCGGTCTCCGGCCAATATTTAGCTTTAGATGAAATTTACCACCCAAT
T AGAGCTGCATTCCCAAACAACTCGACTCGTCGAAGGAGCTTCACACGGG
CGCGGCCACCCCATCCCAGACGGGATTCTCACCCTCTATGACGGCCCGT
TCCAGGGCACTTAGATGGGGGCCGTTCCCGAAGCATCCTCTGCAAATTA
CAATGCGGACCCCGAGGGGGCCAGCTTTCAAATTTGAGCTCTTGCCGCT
TCACTCGCCGTTACTAGGGCAATCCCGGTTGGTTTCTTTTCC
